# Fecal microbiota transplantation in a child with severe ASD comorbidities of gastrointestinal dysfunctions—a case report

**DOI:** 10.3389/fpsyt.2023.1219104

**Published:** 2023-08-17

**Authors:** Cong Hu, Tianyi He, Biao Zou, Heli Li, Jinzhu Zhao, Chen Hu, Jinru Cui, Zhihua Huang, Sainan Shu, Yan Hao

**Affiliations:** ^1^Division of Child Healthcare, Department of Pediatrics, Tongji Hospital, Tongji Medical College, Huazhong University of Science and Technology, Wuhan, China; ^2^Department of Pediatrics, Tongji Hospital, Tongji Medical College, Huazhong University of Science and Technology, Wuhan, China

**Keywords:** fecal microbiota transplantation (FMT), autism (ASD), gastrointestinal dysfunctions, metagenomic sequencing, case report

## Abstract

Autism spectrum disorder (ASD) is a neurodevelopmental disorder defined by social communication impairments and restricted, repetitive behaviors. In addition to behavioral interventions and psychotherapies, and pharmacological interventions, in-depth studies of intestinal microbiota in ASD has obvious abnormalities which may effectively influenced in ASD. Several attempts have been made to indicate that microbiota can reduce the occurrence of ASD effectively. Fecal microbiota transplantation (FMT) is a type of biological therapy that involves the transplant of intestinal microbiota from healthy donors into the patient’s gastrointestinal tract to improve the gut microenvironment. In this case report, we describe a case of child ASD treated by FMT. The patient have poor response to long-term behavioral interventions. After five rounds of FMT, clinical core symptoms of ASD and gastrointestinal(GI) symptoms were significantly altered. Moreover, the multiple levels of functional development of child were also significantly ameliorated. We found that FMT changed the composition of the intestinal microbiota as well as the metabolites, intestinal inflammatory manifestations, and these changes were consistent with the patient’s symptoms. This report suggests further FMT studies in ASD could be worth pursuing, and more studies are needed to validate the effectiveness of FMT in ASD and its mechanisms.

## 1. Introduction

Autism spectrum disorder (ASD) is a neurodevelopmental disorder defined by social communication impairments and restricted, repetitive behaviors (1). The worldwide prevalence of ASD has increased, across all 11 Autism and Developmental Disabilities Monitoring (ADDM) sites in the United States, 1/36 have been estimated to have ASD 8 years old children reported by the American Centers for Disease Control and Prevention (CDC) in 2020, and incidence and median age varied widely from site to site, causing a great economic and social burden. And in recent years, the incidence rate is still gradually increasing (2). The prevalence of ASD in children aged 6–12 years in China is 0.7%, and the overall number of cases exceeds 10 million (3). From a pathogenesis perspective, ASD may be caused by a combined interaction of abnormal genetic factors, exposure to adverse environmental factors, leading to neurological abnormalities (4). However, there is no effective and specific treatment for ASD, the current treatment of ASD mainly focuses on various forms of educational intervention training. But it is not enough for severe ASD to get a significant improvement in core symptoms (5). Therefore, new approaches for ASD treatment are still being explored.

Children with ASD often experience one or more comorbidities, including attention deficits and hyperactivity, intellectual disability, language delay, and GI dysfunction (5). One of the most significant comorbidities in patients with ASD is GI dysfunction. It is worth noting that between 23 and 70% of patients present with associated symptoms (6). Many individuals with ASD experience significant GI dysfunctions, including altered bowel habits and chronic abdominal pain, which often coincide with their neurological alterations (7). Researchers have shown that there is a strong correlation between the severity of ASD and GI symptoms (8). The fecal microbial imbalance has been extensively studied in recent years. Microbial dysbiosis has been widely recognized as a critical factor in the development of various diseases, including neuroimmune and neurobehavioral conditions (9). Detailed examination of microbiota showed that there is a strong connection between the microbial imbalance in children and ASD development. The gut microbiome has played a crucial role in the bidirectional gut-brain axis that integrates the gut and central nervous system (CNS) activities (10). For example, the gut microbiome could affect the nerves of the brain in different ways, including the neuroendocrine, immune system, and metabolites produced by intestinal bacteria and barrier system, finally changing the behaviors (10, 11). The microbiota-gut-brain (MGB) axis has been extensively studied in animal models, and it is clear that alterations in the composition of microbiota influenced neurological and behavioral outcomes. There are some studies suggested that microbial intervention could improving ASD-associated symptoms, such as antibiotics (12), and FMT (13, 14), and probiotics (15, 16). Fecal microbiota from ASD patients could induce ASD-like behavior in mice, and intervention with the microbiota could improve symptoms (17). Therefore, changing the gut microbiota of children with ASD may be an effective means of improving the symptoms of autism spectrum disorders.

Fecal microbiota transplantation (FMT) is a direct treatment method that attempts to restore healthy microbiota composition and function to patients by transplanting feces from a healthy donor into their gastrointestinal tract. FMT also benefits the intestinal barrier, altering the inflammatory response, regulating immunity, and treating some specific intra-intestinal and extra-intestinal diseases (18). However, only a few studies have been published on FMT treated for ASD, and the direct association between microbiome and ASD is still limited. Given the successful observation that FMT can successfully alleviate ASD, this case reports a case of FMT in a child with severe symptoms who did not respond to continuously many years of comprehensive behavioral interventions.

## 2. Materials and methods

### 2.1. Patient case

We present a case study of a 7-year-old female child who began to say the first words at the age of 1 year and 5 months, could speak simple sentences at the age of 2, and showed obvious regression of language ability at the age of 2 years and 6 months, and showed the phenomenon of not responding to people and not looking at people. The ABC scale was examined in the hospital at the age of 2 years and 10 months, the score was 71, and the patient was clinically diagnosed with ASD. At the age of 4, she stopped talking, did not play with other children, and exhibited apparently stereotypical behaviors such as repeatedly tapping objects and laughing pointlessly. There is an obvious phenomenon of picky eating in eating habits. The Chromosome examination and tandem mass spectrometry showed no abnormality. At the age of 6, there were squinting eyes, waving hands, screaming, tapping hands on the head, and other meaningless behaviors. Later, at the age of 7, she was continuing rehabilitation training after the ASD diagnosis by DSM-5 and ADOS-2 in our hospital in 2020, but the effect was unsatisfactory. Due to the COVID-19 epidemic, training is often spaced out, which has had a significant impact on the treatment of children. In June 2020, parents agreed to implement FMT and conducted a comprehensive evaluation in our hospital, including Autism Behavior Scale (ABC), Pediatric Autism Assessment Scale (CARS), Social Response Scale (SRS), Checklist for Autism in Toddlers-23 (CHAT-23), Autism Treatment Assessment Scale (ATEC), and the Children Neuropsychological and Behavioral Scale-Revision 2016 (CNBS-R2016).

### 2.2. Ethical approval

This work has been carried out in accordance with The Code of Ethics of the World Medical Association (Declaration of Helsinki). The Committee of Tongji Medical College of Huazhong University of Science and Technology [(2020) (S290)], approved the study. We gained written informed consent from the minor(s)’ legal guardian, for any potentially identifiable images or data in the publication.

### 2.3. FMT procedure

#### 2.3.1. Fecal microbiota preparation

We selected healthy donors of the same age as the patient, because the species and abundance of fecal bacteria would change with age (19). Fresh feces were collected from the donor, quickly placed in anaerobic bags, and transported to the fecal bacteria transplantation operation room, and the transplantation was completed within 1–2 h. Extraction of fecal bacteria transplant suspension: each gram of healthy feces was diluted with 5 mL of normal saline, mixed thoroughly, filtered with three layers of sterile gauze twice to obtain fresh fecal bacteria solution, and 30–50 mL of sterile injection was extracted. Excess part could be frozen and stored at −80°C after adding glycerol to the fecal bacterial solution.

#### 2.3.2. Fecal microbiota transplant

**Figure 1:**
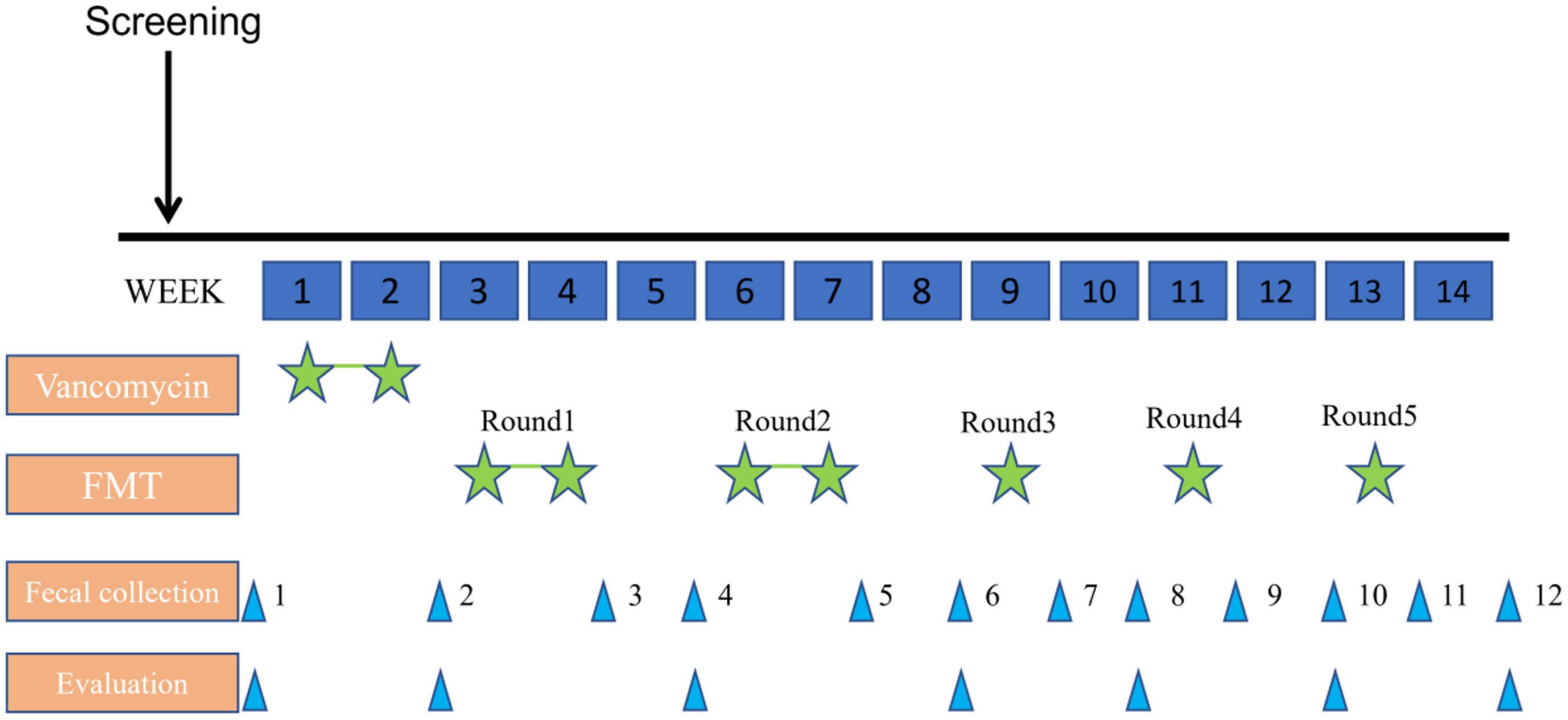
Timeline with relevant timepoints from the episode of study and evaluation.

The patient was given 40 mg/kg/day vancomycin orally for 14 days for colon cleansing before transplantation (oral administration was taken in four divided doses a day). The transplant period was divided into five rounds, each round was separated by a rest period of 1 week and lasted for 3 months in total. For the first time, a colonoscopy was performed to examine the entire colon and distal ileum after standard bowel preparation(fasting for 8 h, water prohibition for 4 h, taking compound polyethylene glycol electrolyte powder). The fecal fluid was perfused from the end of the ileum through an electronic colonoscope, and the fecal fluid was 5 mL/kg each time (80 mL of bacterial solution was used). By transplanting the fluid at the proximal end, the fluid moves distally with the intestinal peristalsis, which will bring the fluid in full contact with the colonic mucosa. After the transplantation, the child slept for 30 min. The rest of the treatment was injected into the child’s digestive tract by enema. After each transplant, the evaluation was performed by ABC, CARS, ATEC, SRS, and CNBS-R2016 (Figure 1).

### 2.4. Microbiological and metabolomics studies

Collect 3–5 g of fecal samples each time in the sterile sampling box, stored at the −80°C. Total DNA was extracted from the stool samples. PCR amplification by using TransGen AP221-02:TransStart Fastpfu DNA Polymerase, and ABI GeneAmp® 9700 was used. The PCR products were detected and quantified using the QuantiFluor™-ST Blue fluorescence quantification system (Promega). Miseq sequencing was performed using the TruSeqTM DNA Sample Prep Kit. The PE reads obtained by Misseq sequencing were first spliced according to the overlap relationship, and the sequence quality was qualitatively controlled and filtered. OTU clustering analysis and species classification analysis were performed after the samples were distinguished. Taxonomic analysis of OTU representative sequences with 97% similar levels was performed by the Ribosomal Database Project (RDP) Classifier, a naïve Bayesian classifier in the http://www.drive5.com. The function of the flora is annotated via http://picrust.github.io/picrust/. The sample diversity was estimated by Bray–Curtis dissimilarity in PcoA， and the data illustration was processed by http://www.ehbio.com/ImageGP/.

## 3. Results

### 3.1. Variation in autism assessment scale data

**Figure 2:**
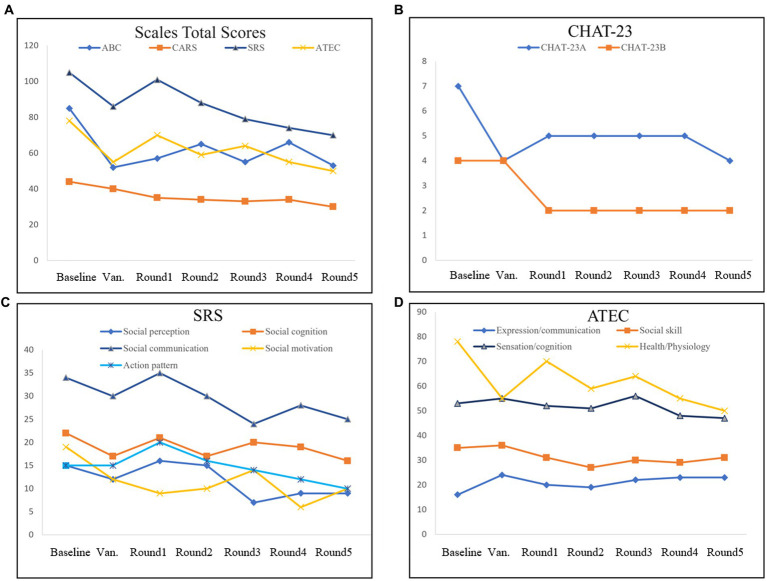
Evaluation of the scales in FMT. The Scales Total Scores **(A)**, including Autism Behavior Scale (ABC), Autism Assessment Scale (CARS), Social Response Scale (SRS), and Autism treatment evaluation checklist (ATEC). Positive items in Checklist for Autism in Toddlers-23 (CHAT-23; **B**). Evaluation checklist details in SRS **(C)**. Evaluation checklist details in ATEC **(D)**.

At the end of all five transplant rounds, the scores of the CARS, ATEC, and SRS gradually decreased, especially after the cleansing phase of vancomycin. The overall ABC score showed a downward trend with some fluctuations (Figure 2A). CHAT-23 is done by the guardian and professional, judging the playing habits and behaviors; the behavior and response to some stimuli, the total score reflects the severity of the illness. The number of positive CHAT-23 items has decreased significantly since one round of treatment (Figure 2B). The total score of SRS reflects the severity of impaired social ability in ASD patients and can be refined into Social perception, social cognition, social communication, and social motivation, a total of four aspects. It can be found that the SRS scores of children will fluctuate slightly after FMT, but the overall downward trend will be obvious after the end of treatment, which indicates the improvement of children’s social ability (Figure 2C). The ATEC scale includes the core symptoms and developmental levels of children with ASD, and its Health/Physiology part can also reflect the diet and sleep problems of children with ASD to some extent (20). Multiple part scores of ATEC decreased after treatment. All of the data indicate that the social behavior of his child has improved significantly after FMT treatment (Figure 2D).

### 3.2. Changes in child development scale data

**Figure 3:**
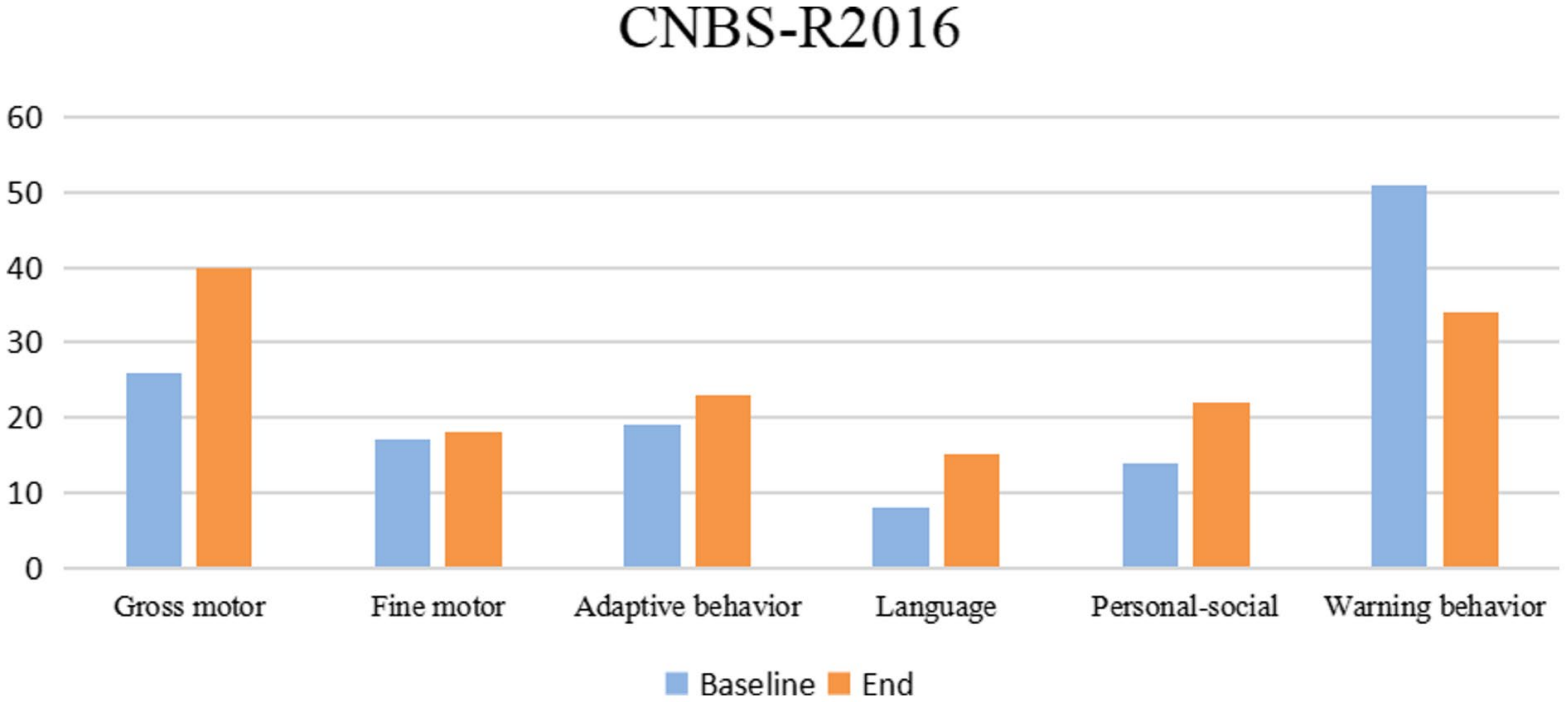
Children’s Neuropsychological and Behavioral Scale—2016 Revision (CNBS-R2016) scores of case before and after FMT treatment.

Children Neuropsychological and Behavioral Scale-Revision 2016 is a widely used developmental assessment tool for children aged 0–6 years in China (21). In the CNBS-R2016 assessment of this child, we can find significant progress in multiple areas after treatment, including gross motor, adaptive behavior, language, and personal-social. Although the scores are still lower than that of typical children (the score should be higher than 85). Besides, the score value decreased significantly in the warning behavior which indicates the severity of ASD. The decrease in the warning behavior score reflected the improvement of the children’s ASD social disorder and stereotyped behavior (Figure 3). There are some details in the different areas. In the Gross motor and Fine motor, movement coordination has improved compared to the previous period, manifested in walking and running, and jumping. Before treatment, she was mainly held by my mother or grandmother, and rarely walks on the ground. After treatment, she could walk or run when going out. In terms of language, children begin to speak the language on the fifth day of the bowel clearing period, calling them “Mom” once a day, and gradually increasing in time. Gradually started to say “No.” During the second transplant period, she could say some simple nouns. During round 3, she would like to say simple sentences “Mom, love you.” At the end of the treatment, she is not only willing to call her mother every day, and constantly say “I love you,” but also occasionally use “I want” to express her needs. In addition, body language has also become meaningful, with movements such as shaking/nodding/waving her hands consistent with the oral expressive meanings. In addition to language development, children have significantly increased emotional communication and begin to observe and respond to parental expressions, such as consolation. Those data and clinical manifestations support that there is a significant improvement in multiple functional areas of the child after FMT treatment.

### 3.3. Changes in intestinal structure

**Figure 4:**
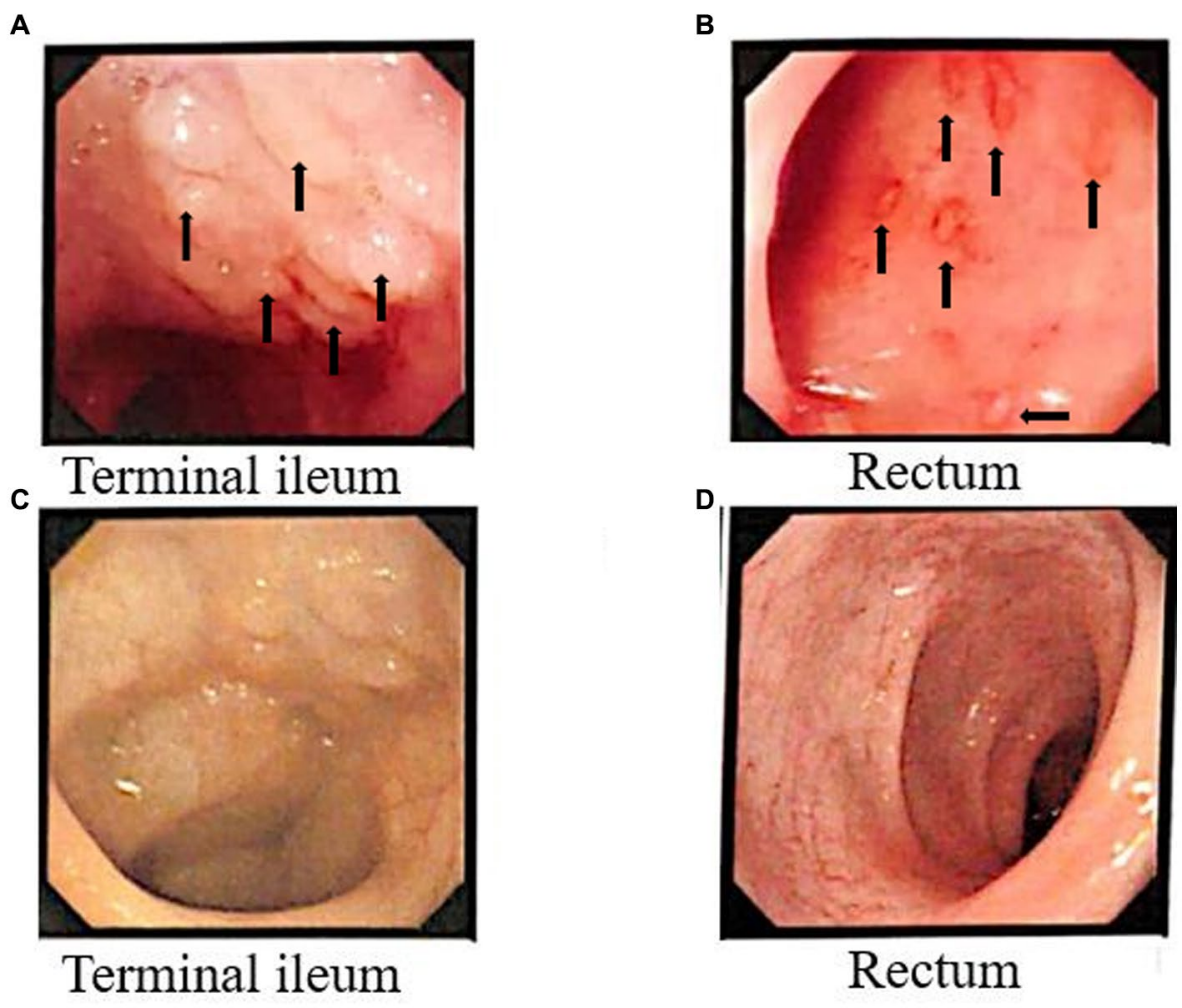
Colonoscopy results before and after FMT. Before FMT, the arrows indicate multiple granulomatous changes in the Terminal ileum **(A)**, and in the rectum **(B)** the arrows indicate bleeding sites accompanied by inflammatory changes. Terminal ileum **(C)** and rectal **(D)** surface results after FMT.

Before FMT, the patient underwent electronic gastroscopy and colonoscopy, and it was found that the end of the ileum was scattered with granular hyperplasia, and the rectum was scattered with shallow ulcers covered with white moss. Pathological findings showed chronic ileus and multiple granulomas with unclear boundaries in the mucosa. Immunohistochemical staining was performed for CD68 (focal +), CD163 (scattered +), and S-100 (scattered +). After fecal bacteria transplantation, only a few granular hyperplasias were observed at the end of the ileum. The other intestinal mucosa was smooth, and the rectal ulcer was healed under the microscope (Figure 4).

### 3.4. Changes in the composition of the microbiota

**Figure 5:**
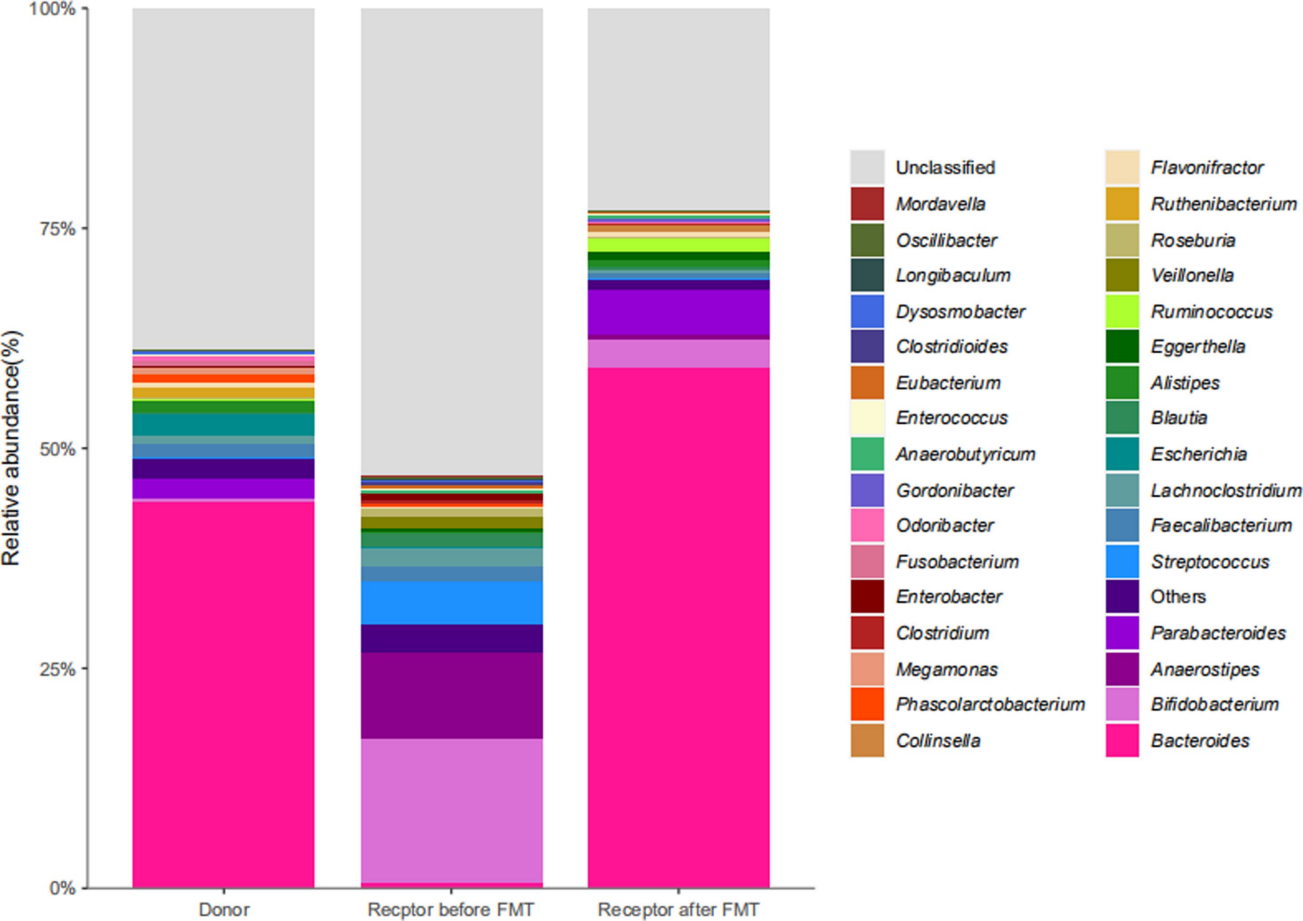
Metabolite profiles before and after fecal microbiota transplantation.

**Figure 6:**
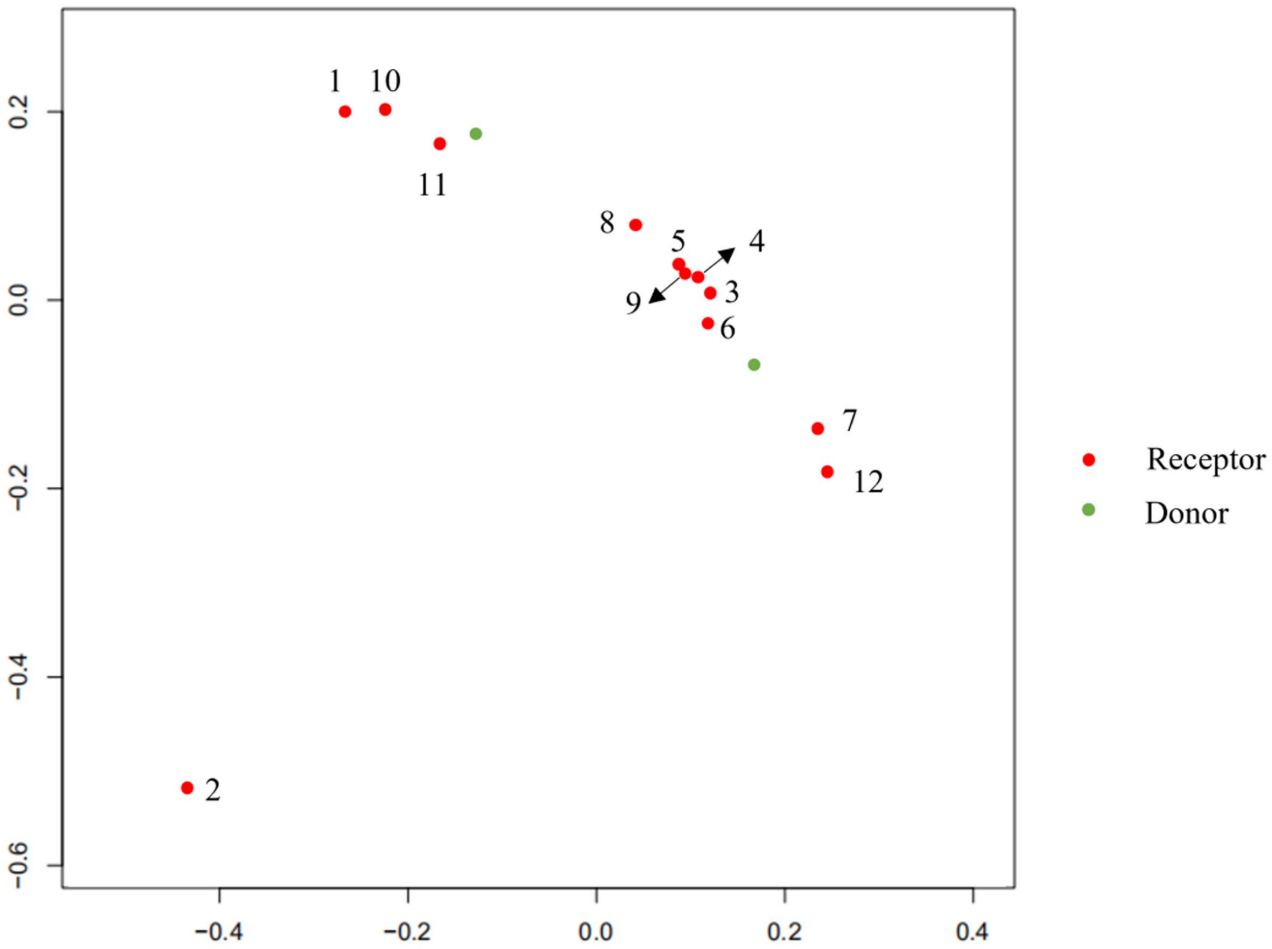
PCA analysis at different periods of transplantation in Doner and Receptor.

To explore the changes in the child’s flora after treatment, we analyzed the composition and metabolic function through microbiota analysis. Before FMT, the intestinal microbiota diversity and proportion of Bacteroides were significantly lower than that of the donor. After FMT, the abundance of Bacteroides increased significantly, as did Ruminococcus. On the contrary， Bifidobacterium, Anaerostipes, Streptococcus, and Faecalibacterium showed significant declines after transplantation (Figure 5). Furthermore, the production capacity of short-chain fatty acids (SCFAs) was weak (Table 1), which is important for behavioral development (6). After FMT, the diversity of the flora was significantly improved, as we can see the diversity scores increased from 22 to 92, and the diversity of flora composition was closer to the donor (Figure 6). Surprisingly, the proportion of the Bacteroides was significantly increased, which may be a bacterium that plays an important role (Figure 5). The FMT also improved the production capacity of SCFAs from weak (0%) to normal (20%). In addition, the dietary bias of the children was similar to the donor (Table 1).

**Table 1:** Characteristics of flora and diet.

	Donor	Pre-transplant recipients	Post-transplant recipients
Diversity of flora	High (78 points)	Normal (22 points)	High (92 points)
Production capacity of SCFAs	Normal	Weak	Normal
Production capacity of butyric acid	Normal (35%)	Weak (0%)	Normal (20%)
Dietary bias	High in protein and meat	High fiber	High in protein and meat

## 4. Discussion and conclusion

This study confirmed that the child with ASD Autism co-exists with GI symptoms and has made significant progress in multiple abilities after FMT treatment. In clinical observation, it could be found that the improvement in eating and defecation appears the earliest, in the intestinal clearing period of antibiotics. At the end of the transplant she could eat out. The change in behavior associated with vancomycin is similar to previous studies, suggesting the importance of vancomycin in FMT (22). There were some improvements observed in the initial 1–2 treatment round in eye contact, olfactory abnormalities, and partially stereotyped behaviors. Social ability and emotional changes mainly partly appropriate emotional expression. In terms of motor and self-care ability, children can jump on both feet from the one round to the 4–5 treatment round, which is rarely held by their parents, and is more willing to walk or run by herself. In terms of language, she could call her mom from the first round, moving forward to say “Mom, I love you” in the third round. Before each transplant round, the subjects showed no abnormalities in serum, urine, or stool routine tests, with no severe rash, vomiting, diarrhea, abdominal pain, fever, abdominal distension, and emotional problems. Above all, this case report suggests that FMT can improve the social viability of autistic children and improve living quality.

Next, to explore the changes in the flora of children after treatment, Chen et al. found that FMT alleviated behavioral abnormalities and chemokine defects in mouse models of ASD by performing gut microbiota transplantation in a mouse model of ASD induced by maternal immune activation(MIA). In addition, some key unique taxa in the composition of the ASD gut microbiome have also changed (17). FMT from donors of children with autism led to colonization of ASD-like microbiota and autistic behavior. These alterations are closely associated with the expression levels of proinflammatory factors IL-1β, IL-6 in the brain and intestine (23). Elaine et al. (24) using oral treatment of MIA offspring with the human commensal *Bacteroides fragilis* corrects gut permeability, alters the microbial composition, and ameliorates defects in communicative, stereotypic, anxiety-like, and sensorimotor behaviors. Ruminococcus are mucosa-associated bacteria linked with gastrointestinal disease, which have been confirmed that altered in ASD patients (25). In the mice models, bifidobacteria could play a role in the pathogenesis of ASD by modifying the correction of oxidative stress, and restoration of depleted GABA (26). We are pleased to be able to find alterations in these flora in the treatment, unfortunately, the abundance changes may be biased because this case involved only one patient. Those results are consistent with the change in *Bacteroides fragilis* abundance in this case. There is another research that indicates that gut microbiota from individuals with ASD is sufficient to promote altered behaviors in Mice. Furthermore, treating an ASD mouse model with candidate microbial metabolites which includes taurine and 5AV improves behavioral abnormalities and modulates neuronal excitability in the brain (27). Daniel et al. (28) determined that SCFAs, microbial-derived bacterial fermentation products, could regulate microglia homeostasis and play an important role in the onset of autism. In this case, the ability of the microbiota to produce SCFAs, particularly butyric acid, was significantly increased after FMT, which may be an important cause of induced behavior change. This also suggests that behavioral improvement led by SCFAs may be a potential new therapeutic direction in ASD. Significant analysis and discussion were presented by Yap et.al about the relationship between this microbiome and diet, who hold an idea that microbiome differences in ASD may reflect dietary preferences that relate to diagnostic features (29). Dan et al. revealed some different neurotransmitters which are associated with certain specific bacteria in normal individuals and ASD. The neurotransmitters include serotonin, dopamine, histidine, and GABA which play a critical role in neurological development (30). These substances may pass through the leaky gut and blood–brain barrier, and eventually the effects on the functional brain areas associated with ASD. For example, social cognition of ASD has been shown to be altered in cerebellar gray matter (31). To sum up, factors caused by changes in the flora, such as immunity, metabolites, and neurotransmitters, may collectively influence the etiology of ASD by exacerbating the severity of symptoms.

In conclusion, the treatment effect of this case is very satisfying to patients and doctors, however, there are still some deficiencies and reflections. Firstly, the clinical trials of FMT for ASD are still inadequate, especially, the long-term effects of this treatment have not been adequately clarified. Secondly, FMT offers a new dawn for the treatment of ASD, but the direct contribution of the microbiota to the pathophysiology and behavioral outcomes of ASD still needs to be further explored.

## Data availability statement

The original contributions presented in the study are included in the article/supplementary material, further inquiries can be directed to the corresponding authors.

## Ethics statement

The studies involving humans were approved by The Committee of Tongji Medical College of Huazhong University of Science and Technology [(2020) (S290)], approved the study. The studies were conducted in accordance with the local legislation and institutional requirements. Written informed consent for participation in this study was provided by the participants’ legal guardians/next of kin. Written informed consent was obtained from the individual(s), and minor(s)’ legal guardian/next of kin, for the publication of any potentially identifiable images or data included in this article.

## Author contributions

CoH and TH wrote the first draft of the manuscript and coordinated and supervised data collection. BZ and ZH performed all FMT. JZ, ChH, HL, SS, and YH substantially contributed significantly to the design of the experiment and critically reviewed and revised manuscripts. All authors contributed to the article and approved the submitted version.

## Funding

This work was supported by Key Project of Independent Innovation Research Fund of Huazhong University of Science and Technology (Grant Number 2017KFYXJJ100).

## Conflict of interest

The authors declare that the research was conducted in the absence of any commercial or financial relationships that could be construed as a potential conflict of interest.

## Publisher’s note

All claims expressed in this article are solely those of the authors and do not necessarily represent those of their affiliated organizations, or those of the publisher, the editors and the reviewers. Any product that may be evaluated in this article, or claim that may be made by its manufacturer, is not guaranteed or endorsed by the publisher.
